# The Relationship between P-Selectin Polymorphisms and Thrombosis in Antiphospholipid Syndrome: A Pilot Case-Control Study

**DOI:** 10.4274/tjh.2013.0091

**Published:** 2014-12-05

**Authors:** Nilüfer Alpay, Veysel Sabri Hançer, Burak Erer, Murat İnanç, Reyhan Diz-Küçükkaya

**Affiliations:** 1 Istanbul University İstanbul Faculty of Medicine, Department of Internal Medicine, Division of Rheumatology, İstanbul, Turkey; 2 Istanbul Bilim University Faculty of Medicine, Department of Medical Biology and Genetics, İstanbul, Turkey; 3 İstanbul Bilim University Faculty of Medicine, Department of Internal Medicine, Division of Hematology, İstanbul, Turkey

**Keywords:** P-selectin polymorphisms, Thrombosis, Antiphospholipid syndrome

## Abstract

**Objective:** The selectins are cell adhesion molecules that mediate the interactions among leukocytes, activated platelets, and endothelial cells. We aimed to investigate whether P-selectin polymorphisms are associated with thrombosis in patients with antiphospholipid syndrome (APS).

**Materials and Methods:** The diagnosis and classification of APS were based on the report of an international workshop. Genomic DNA was extracted from citrated blood samples of all subjects. Three single nucleotide polymorphisms associated with the P-selectin coding region (S290N, c.1087G>A; N562D, c.1902G>A; T715P, c.2363A>C) were assessed.

**Results:** There were 26 APS (65%) patients with thrombosis. The number of patients without thrombosis was 14 (35%). The frequency of the N562D-DN genotype was significantly higher in patients with APS than in healthy controls (p=0.003). The frequency of this genotype was significantly higher in patients with APS with thrombosis compared with patients with no thrombosis (p=0.03). The N562D-NN genotype was found at a higher frequency in patients with APS than in healthy controls (p=0.004).

**Conclusion:** Our results suggest that the N562D polymorphism of the DN genotype of P-selectin is associated with an increased risk of thrombosis in patients with APS.

## Abstract (tr)

**Amaç:** Hücre adezyon molekülü olan selektinler, lökositlerle, aktive plateletler ya da endotel hücreleri arasındaki etkileşime aracılık eder. Bu çalışmada antifosfolipid sendromu (AFS) hastalarında tromboz riski ile P-selektin polimorfizmleri arasındaki ilişkinin araştırılması amaçlanmaktadır.

**Gereç ve Yöntemler:** AFS tanısı “International Workshop” tanı ve sınıflandırma kriterleri ile konulmuştur. Hastaların periferik kan örneklerinden DNA elde edilmiştir. P-selektin gen bölgesiyle ilişkili üç tane tek nükleotid polimorfizmi (S290N, c.1087G>A; N562D, c.1902G>A; T715P, c.2363A>C) araştırılarak genotipleri belirlenmiştir.

**Bulgular:** Hasta grubunda 26 trombozlu (%65) AFS olgusu ve 14 (%14) trombozu olmayan AFS olgusu yer almaktadır. Hasta ve kontrol grubu kıyaslandığında incelenen polimorfizmlerden N562D-DN genotipi hasta grubunda anlamlı olarak yüksek bulunmuştur (p=0,003). Yine hasta grubuna bakıldığında trombozlu AFS grubunda trombozu olmayan AFS grubuna kıyasla N562D-DN genotipine anlamlı olarak sık rastlanmıştır (p=0,03). N562D NN genotipi ise kontrol grubunda hasta grubuna oranla daha yüksek sıklıktadır (p=0,004).

**Sonuç:** P-selektin N562D polimorfizmi DN genotipi primer AFS hastalarında tromboz riski ile ilişkili olduğu söylenebilir.

## INTRODUCTION

Antiphospholipid syndrome (APS) is an autoimmune disease characterized by pregnancy morbidity and arterial-venous thrombosis in the presence of antiphospholipid antibodies (aPLAs) [1]. Although the relationship between aPLAs and thrombosis is known, the mechanisms of thrombosis in APS has not been fully elucidated. Selectins, which are cell adhesion molecules, mediate the interactions among leukocytes, activated platelets, and endothelial cells. P-selectin, which can be identified as a soluble form in plasma, intercedes in the attachment and rolling of leukocytes on activated endothelial cells and is involved in the recruitment of leukocytes to thrombi [2,3]. Novel data suggest that the levels of soluble P-selectin (sP-selectin) are increased in APS patients with thrombosis [4]. In this study, we aim to investigate whether P-selectin polymorphisms are associated with thrombosis in patients with APS.

## MATERIALS AND METHODS

**Patients and Controls**

Forty adult patients with APS and 40 healthy subjects with no history of thrombosis or autoimmune disease were included in the study. The history of disease, physical examination, and screening of lupus anticoagulant (LA) and serum anticardiolipin IgG and IgM levels were assessed for all patients. The diagnosis and classification of APS were based on an international consensus statement [1]. The subjects participating in the study had no LA and/or serum anticardiolipin-related systemic diseases or risk factors such as hypertension or hyperlipidemia for thrombosis.

LA was diagnosed using activated partial thromboplastin time, kaolin clotting time, and Russell’s viper venom test according to published criteria [5]. IgG and IgM anticardiolipin antibodies were determined by enzyme-linked immunosorbent assay [6], and levels equal to or greater than 4 standard deviations were regarded as positive. The anticardiolipin antibody and LA tests were repeated 3 months after the first determination. APS patients with thrombosis had arterial and/or venous thrombosis as well as aPLA positivity. aPLA-positive patients with no thrombosis suffered from either first trimester fetal losses or thrombocytopenia and had persistently positive aPLA test results but no thrombotic complications over at least 3 years of follow-up.

The study protocol was approved by the local ethics committee, and written and signed informed consent was obtained from all participants.

**Genotyping**

Genomic DNA was extracted from citrated blood samples. Three single nucleotide polymorphisms associated with the P-selectin coding region (S290N, c.1087G>A; N562D, c.1902G>A; T715P, c.2363A>C) were assessed. Polymerase chain reaction (PCR) was done in a total volume of 25 µL containing 2 U of Taq DNA polymerase (Fermentas), 2 mmol/L MgCl2, 0.2 mmol/L of each dNTP, 2.5 µL of 10X PCR buffer, and 50 ng of genomic DNA. 

Allele specific primers were used in the following concentrations: 15 pmol of 290N-R (5’-TAAATGAATTCAGTCCATGGTTCCTACAT-3’), 5 pmol of 290S-R (5’-CACAGTCCATGGTTCCTTGAC-3’), 11 pmol of 290common (5’-TGTGTGGCTTTTCTCCTTTC-3’), 2 pmol of 562D-R (5’-ATTGCCCTACCAGCTTAAAGCCG TAGTC-3’), 7 pmol of 562N-R (5’-CTCCAGCTTAAAGCCGTTCTT-3’), 10.5 pmol of 562common (5’-TGAATATATAAGTGA ATGAACTTTGTG-3’), 3.5 pmol of 715P-R (5’-CCT GCT TGATAG GTT GCC ACG GAA GG-3’), 8 pmol of 715T-R (5’-GCAGGT TGG CAC GGT TGT-3’), and 9 pmol of 715common (5’-CTGTGA AAT GCT CAG AAC TAC ATG-3’). PCR amplification was carried out in a GeneAmp PCR System 9700 Thermo Cycler (Applied Biosystems, USA) using 36 cycles of 94 °C for 25 s, 57 °C for 25 s, and 72 °C for 25 s. PCR products were separated on agarose gels and stained with ethidium bromide. The PCR products were 115, 205, and 182 bp long for S290N, N562D, and T715P, respectively.

## STATISTICAL ANALYSIS

Data are expressed as mean ± SD, number (%), or median (range). Test statistics were computed using the Mann-Whitney U test and the Kruskal-Wallis test. The chi-square test and odds ratio were used to calculate the 95% confidence intervals. Correlation coefficients and significance were calculated by Spearman’s test to assess the differences between groups. For all tests, a 2-tailed p-value of <0.05 was considered statistically significant. Statistical analyses were performed using the software package SPSS 15 running on Windows NT.

## RESULTS

There were 26 APS (65%) patients with thrombosis, 12 (46%) of which cases involved veins, 10 (38%) arteries, and 4 (15%) veins and arteries together. The number of APS patients without thrombosis was 14 (35%). The mean age of patients (80% female) was 39.4±9.5 years. The characteristics of patients are seen in Table 1. The frequency of the N562D-DN genotype was significantly higher in patients with APS than healthy controls (p=0.003). The frequency of this genotype was significantly higher in patients with APS with thrombosis compared to patients without thrombosis (p=0.03). The N562D-NN genotype was found at a higher frequency in patients with APS than in healthy controls (p=0.004). The frequency of the N562D-NN genotype was not different between patients without thrombosis and control subjects (p=0.21). On the other hand, S290N and T715P polymorphisms were not different between patient and control groups (Tables 2 and 3). There was no relationship between aPLA, thrombocytopenia, or pregnancy loss and any polymorphism.

## DISCUSSION

P-selectin is expressed on activated platelets and endothelial cells. P-selectin glycoprotein ligand-1 (PSGL-1) is found in neutrophils and monocytes, and these are from microparticles. Connecting P-selectin/PSGL-1 activated leukocytes for endothelium rolling, and providing the release of tissue factor initiates thrombosis [7]. Previous studies suggest that sP-selectin levels increased in patients with thrombosis after a finding of the association of P-selectin and thrombosis in an animal model [8,9,10]. Additionally, P-selectin polymorphisms were detected in patients with thrombosis, but not always together with high sP-selectin levels. High sP-selectin levels were detected in patients with systemic lupus erythematosus and APS [11,12]. According to other studies, P-selectin polymorphisms play a role in the pathogenesis of systemic lupus erythematosus [13,14].

On the other hand, the relationship between polymorphism of PSGL-1 and ischemic cerebrovascular disease was shown previously [15]. Roldan et al. demonstrated that short alleles of PSGL-1 protect against cardiovascular disease [16]. Additionally, the relationship PSGL-1 VNRT polymorphisms and risk of thrombosis in APS patients was shown by Diz-Kucukkaya et al. [17]. The relationship between P-selectin polymorphism and thrombosis in APS patients was shown for the first time in our study. The c.1087G>A, c.1902G>A, and c.2363A>C polymorphisms lead to S290N, N562D, and T715P P-selectin gene variations, respectively. These variations are in the genetic region encoding the repeated part of the P-selectin gene and may be effective in binding P-selectin to PSGL-1. Therefore, our data are valuable in order to determine risk factors other than traditional ones for thrombosis in APS despite the fact that we did not estimate sP-selectin levels.

In conclusion, our results suggest that the N562D polymorphism DN genotype of P-selectin is associated with an increased risk of thrombosis in patients with APS. The NN genotype of the same polymorphism might be protective against thrombosis in those patients. The effect of N562D polymorphism on sP-selectin levels will be studied in future work.

**Conflict of Interest Statement**

The authors of this paper have no conflicts of interest, including specific financial interests, relationships, and/ or affiliations relevant to the subject matter or materials included.

## Figures and Tables

**Table 1:**
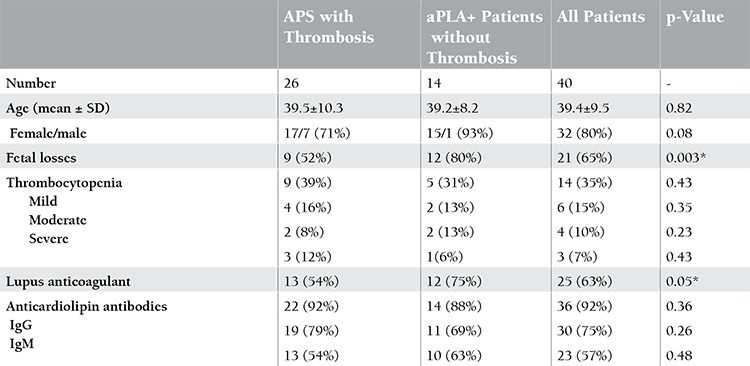
Characteristics of the patients with antiphospholipid syndrome.

**Table 2:**
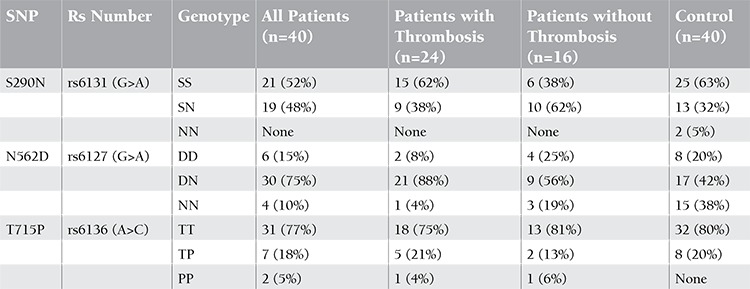
The frequency of S290N, N562D, and T715P polymorphisms in the patients and control groups.

**Table 3:**
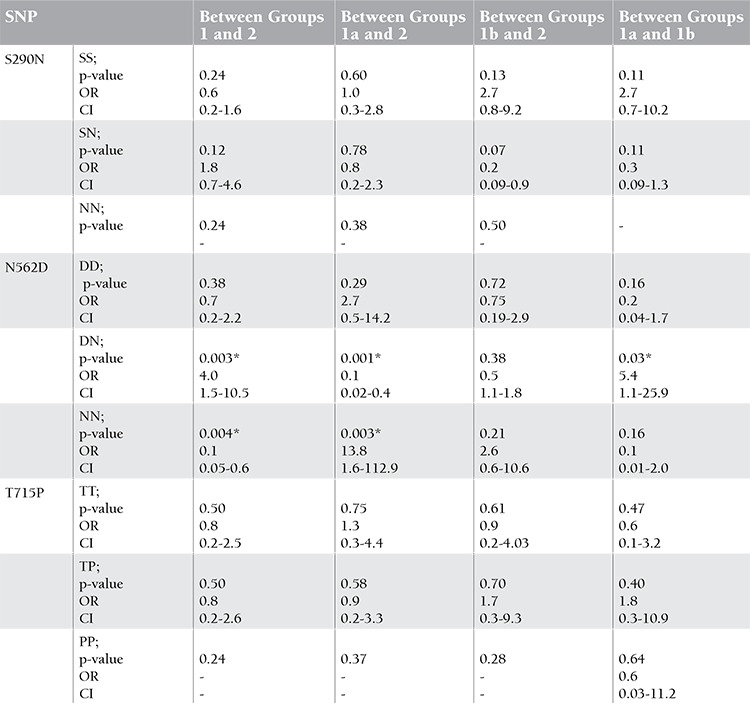
The differences in S290N, N562D, and T715P polymorphisms among groups (group 1: all patients, group 1a: patients with thrombosis, group 1b: patients without thrombosis, group 2: control subjects).
